# Polypharmacy Patterns: Unravelling Systematic Associations between Prescribed Medications

**DOI:** 10.1371/journal.pone.0084967

**Published:** 2013-12-20

**Authors:** Amaia Calderón-Larrañaga, Luis A. Gimeno-Feliu, Francisca González-Rubio, Beatriz Poblador-Plou, María Lairla-San José, José M. Abad-Díez, Antonio Poncel-Falcó, Alexandra Prados-Torres

**Affiliations:** 1 EpiChron Research Group on Chronic Diseases, Aragón Health Sciences Institute (IACS), IIS Aragón, Miguel Servet University Hospital, Zaragoza, Spain; 2 Department of Microbiology, Preventive Medicine and Public Health, University of Zaragoza, Zaragoza, Spain; 3 Red de Investigación en Servicios de Salud en Enfermedades Crónicas (REDISSEC), Carlos III Health Institute, Madrid, Spain; 4 Teaching Unit of Preventive Medicine and Public Health, Aragón Health Sciences Institute (IACS), IIS Aragón, Zaragoza, Spain; 5 San Pablo Health Centre, Zaragoza, Spain; 6 Delicias Sur Health Centre, Zaragoza, Spain; 7 Department of Medicine, Psychiatry and Dermatology, University of Zaragoza, Zaragoza, Spain; 8 Department of Health Wellbeing and Family, Government of Aragón, Zaragoza, Spain; 9 Primary Care Directorate, Aragón Health Service, Zaragoza, Spain; Universidad de Valladolid, Spain

## Abstract

**Objectives:**

The aim of this study was to demonstrate the existence of systematic associations in drug prescription that lead to the establishment of patterns of polypharmacy, and the clinical interpretation of the associations found in each pattern.

**Methods:**

A cross-sectional study was conducted based on information obtained from electronic medical records and the primary care pharmacy database in 2008. An exploratory factor analysis of drug dispensing information regarding 79,089 adult patients was performed to identify the patterns of polypharmacy. The analysis was stratified by age and sex.

**Results:**

Seven patterns of polypharmacy were identified, which may be classified depending on the type of disease they are intended to treat: cardiovascular, depression-anxiety, acute respiratory infection (ARI), chronic obstructive pulmonary disease (COPD), rhinitis-asthma, pain, and menopause. Some of these patterns revealed a clear clinical consistency and included drugs that are prescribed together for the same clinical indication (i.e., ARI and COPD patterns). Other patterns were more complex but also clinically consistent: in the cardiovascular pattern, drugs for the treatment of known risk factors—such as hypertension or dyslipidemia—were combined with other medications for the treatment of diabetes or established cardiovascular pathology (e.g., antiplatelet agents). Almost all of the patterns included drugs for preventing or treating potential side effects of other drugs in the same pattern.

**Conclusions:**

The present study demonstrated the existence of non-random associations in drug prescription, resulting in patterns of polypharmacy that are sound from the pharmacological and clinical viewpoints and that exist in a significant proportion of the population. This finding necessitates future longitudinal studies to confirm some of the proposed causal associations. The information discovered would further the development and/or adaptation of clinical patient guidelines to patients with multimorbidity who are taking multiple drugs.

## Introduction

Multimorbidity, which is defined as suffering from multiple chronic diseases simultaneously, has begun to be considered a major health problem affecting developed countries [1]. In addition to its undesirable consequences for the patient (e.g., poorer quality of life and functional capacity) and health services (e.g., misuse and saturation of services) [2], multimorbidity presents a challenge for the physicians who are responsible for the care of these patients because of the absence of available evidence for the concurrent management of multiple chronic diseases, among other factors. Despite being effective for the individual diseases upon which they are focused, most clinical practice guidelines (CPGs) do not pay enough attention to the presence of different health problems within the same patient [3].

Drug therapy is an area in which this shortage of available evidence is most clearly manifested since the use of multiple disease-focused CPGs might lead to misinterpretations by GPs. In fact, polypharmacy has frequently undesirable consequences, such as increased risk of inappropriate drug use, under-use of effective treatments, medication errors, poor adherence, drug-drug and drug-disease interactions and, most importantly, adverse drug reactions [4]. However, these threats to the safety of patients, which may further compromise their clinical situation, are rarely considered in the development of CPGs [3]. This is of particular relevance in the Spanish healthcare system, where a considerable proportion of pharmaceutical prescriptions are induced by specialists [5,6], who themselves are more subject to use a CPG for each disease than GPs.

According to the European Forum for Primary Care, an important step in producing and/or adapting the available evidence to patients with multimorbidity lies in the consideration of the systematic associations between diseases and drugs [7]. In this regard, within the last five years, there has been an increase in the number of studies aimed at determining patterns of diseases (or multimorbidity) and their underlying pathophysiological mechanisms. One of the most recent studies, which was conducted in adults, revealed the existence of five multimorbidity patterns that the authors name as follows: 1) cardio-metabolic, 2) psychiatric-substance abuse, 3) mechanical-obesity-thyroid, 4) psychogeriatric, and 5) depressive [8]. In addition to a warning about the urgent need for a paradigm shift in the clinical approach to the patient, these studies recommend investigating the existence of patterns of polypharmacy (i.e., common associations between drugs) as both causal and consequent factors of existing disease clusters in the population.

The general objective of this paper is to show the existence of systematic associations in drug prescription and use, which create patterns of polypharmacy. The specific objectives are 1) to describe drugs that constitute these patterns, 2) to estimate the prevalence of these patterns, and 3) to interpret clinically the associations found in each pattern. 

## Methods

A cross-sectional study was conducted based on data obtained from electronic medical records and pharmacy billing records for 79,089 patients who were over 14 years of age and had been seen at least once by their family doctor in 2008. The seven Zaragoza health centres that were included in the study were previously selected based on criteria related to the quality of the collected information [8].

Demographic variables of the age and sex were extracted from patients’ electronic medical records. Data on prescribed and dispensed active ingredients during 2008, and the date of dispensing was obtained from the pharmacy billing records. The active ingredients were coded according to the Anatomical Therapeutic Chemical Classification System (ATC) [9], considering the first three levels of the classification, to facilitate the processing of the data. To ensure the concurrent use of drugs for the same patient and to take account of drugs with a seasonal utilisation, monthly dispensing frequencies for each drug were first analysed, selecting the two months with higher dispensing for the final analysis, namely, January and February.

This study was favourably evaluated by the Clinical Research Ethic Committee of Aragon (CEICA). Written consent by patients was not needed since the study does not involve interventions on individuals, the use of human biological samples, or the analysis of personally identifiable data. Instead, the present work is based on the statistical analysis of anonymous data contained in previously existing databases which were obtained with prior permission from the corresponding entity.

### Statistical Analysis

All of the analyses were stratified by sex and age; the latter variable was grouped into three intervals: 15-44, 45-64, and ≥64. The identification of patterns of polypharmacy was based on exploratory factor analysis, which is one of the methods commonly used to identify patterns of multimorbidity [10]. The strength of this technique is that, in addition to identifying non-random associations between groups of variables, it allows a single variable to be part of different patterns. Drugs with over 1% prevalence in each age and sex group were included in the analysis to yield epidemiologically relevant results.

The factor analysis was based on a tetrachoric correlation matrix, and the factor extraction was performed using the principal factor method. The number of factors to extract was determined using sedimentation graphs (Figure A in File S1) and the clinical evaluation from the different solutions. The adequacy of the sample was analysed using the Kaiser-Meyer-Olkin (KMO) parameter, and the cumulative proportion of the variance as a measure of model fit was obtained. To determine the drugs that belonged to each pattern, those medications with scores higher than 0.30 for each factor were selected (Tables A-F in File S2). The clinical interpretation of the patterns was conducted by three medical doctors (two specialising in family medicine [LGF and FGR] and one specialising in preventive medicine [APT]), one pharmacist [MLS], and one epidemiologist [ACL].

To estimate the prevalence of the individual patterns of polypharmacy, the number of individuals who had a certain pattern was determined, considering that an individual belonged to the pattern if he/she had been dispensed at least three of the drugs included in the pattern. It is understood that the threshold of three drugs dispensed simultaneously could justify, by itself, some of the undesirable effects of polypharmacy [11]. 

The STATA 11.0 software was used for the statistical analyses. 

## Results and Discussion

The study population was composed of 79,089 patients who were over 14 years of age (with a mean age of 47 years), of which 55.03% were women (Table 1). In total, 63.8% of the men aged 15-44 years were taking no medication, a figure that decreased to 9.75% for those men over 64 years of age. The concurrent use of drugs increased with age in both men and women; 70.91% of the men and 80.19% of the women over 64 years of age used more than two medications, and 36.65% and 47.6%, respectively, used over five medications (Table 1).

**Table 1:** Baseline characteristics of the study population.

	**Women**			**Men**		
	**15-44 years**	**45-64 years**	**≥65 years**	**15-44 years**	**45-64 years**	**≥65 years**
	**n (%)**	**n (%)**	**n (%)**	**n (%)**	**n (%)**	**n (%)**
**Age and sex distribution**	18,779 (23.74)	13,807 (17.46)	12,016 (15.19)	15,341 (19.4)	10,725 (13.56)	8,418 (10.64)
**Main clinical conditions registered by GPs**						
**Hypertension**	230 (1.22)	2,347 (17)	6,154 (51.22)	324 (2.11)	2,333 (21.75)	3,709 (44.06)
**Diabetes**	150 (0.8)	718 (5.2)	1,986 (16.53)	170 (1.11)	1,076 (10.03)	1,666 (19.79)
**Dyslipidaemia**	316 (1.68)	1,903 (13.78)	2,095 (17.44)	646 (4.21)	1,879 (17.51)	1,382 (16.42)
**Atherosclerosis**	20 (0.11)	42 (0.3)	74 (0.62)	13 (0.08)	93 (0.87)	178 (2.11)
**Ischemic heart disease**	2 (0.01)	59 (0.43)	270 (2.25)	13 (0.08)	149 (1.39)	314 (3.73)
**Heart failure**	3 (0.02)	22 (0.16)	287 (2.39)	5 (0.03)	31 (0.29)	160 (1.9)
**Anxiety, neuroses**	1,591 (8.47)	1,437 (10.41)	933 (7.76)	731 (4.77)	506 (4.72)	279 (3.31)
**Depression**	571 (3.04)	1,003 (7.26)	857 (7.13)	233 (1.52)	307 (2.86)	218 (2.59)
**Behaviour problems**	437 (2.33)	482 (3.49)	533 (4.44)	262 (1.71)	225 (2.1)	240 (2.85)
**Parkinson’s disease**	---	15 (0.11)	82 (0.68)	---	11 (0.1)	73 (0.87)
**Acute respiratory infection**	640 (3.41)	830 (6.01)	1,127 (9.38)	494 (3.22)	554 (5.16)	856 (10.17)
**COPD**	16 (0.09)	102 (0.74)	231 (1.92)	18 (0.12)	240 (2.24)	744 (8.84)
**Allergic rhinitis**	711 (3.79)	346 (2.51)	175 (1.46)	496 (3.23)	184 (1.72)	124 (1.47)
**Asthma**	439 (2.34)	328 (2.38)	346 (2.88)	336 (2.19)	125 (1.17)	111 (1.32)
**Osteoporosis**	21 (0.11)	769 (5.57)	1,227 (10.21)	8 (0.05)	30 (0.28)	77 (0.91)
**Number of drugs taken simultaneously**						
**0**	10,094 (53.75)	3,977 (28.8)	856 (7.12)	9,788 (63.8)	3,856 (35.94)	821 (9.75)
**1**	2,989 (15.92)	1,931 (13.99)	574 (4.78)	2,129 (13.88)	1,614 (15.04)	709 (8.42)
**2**	2,574 (13.71)	1,939 (14.04)	951 (7.91)	1,712 (11.16)	1,572 (14.65)	919 (10.92)
**≥3**	3,122 (16.62)	5,960 (43.18)	9,635 (80.19)	1,712 (11.17)	3,686 (34.36)	5,969 (70.91)

Seven patterns of polypharmacy were identified in this study, which, depending on the type of disease they are intended to treat, may be classified as cardiovascular, depression-anxiety, acute respiratory infection (ARI), chronic obstructive pulmonary disease (COPD), rhinitis-asthma, pain, and menopause. The first four patterns occurred in both men and women, the following two occurred only in men, and the latter occurred only in women (Table 2). 

**Table 2:** Patterns and prevalence* of polypharmacy by age group and sex.

	**15-44 years**	**Prev. (%)**	**45-64 years**	**Prev. (%)**	**≥ 65 years**	**Prev. (%)**
**Women**	Depression-anxiety	0.53	Depression-anxiety	11.90	Depression-anxiety	37.48
	ARI	8.39	Cardiovascular	3.62	Cardiovascular	8.85
			ARI	7.39	COPD	7.41
			Menopause	0.55		
**Men**	Depression-anxiety	0.78	Depression-anxiety	2.04	Depression-anxiety	0.29
	ARI	3.55	Cardiovascular	10.85	Cardiovascular	24.63
	Rhinitis-asthma	0.09	COPD	5.18	COPD	25.34
			Pain	4.52		

^*^ Numerator: individuals with three or more drugs of the pattern within each age and sex group; Denominator: the entire population within each age and sex group.

A clear clinical consistency was identified in some of these patterns because they encompassed various therapeutic groups with a common clinical indication—as was the case for the ARI or COPD patterns. Other patterns were more complex but also clinically consistent; this was the case of the cardiovascular pattern, which included medications for the treatment of known risk factors (i.e., diabetes, hypertension, or dyslipidemia) along with other medications aimed at preventing complications (e.g., antiplatelet agents). Finally, this study also discovered associations that are difficult to explain based on the available clinical knowledge and that should facilitate the design of future research efforts.

The various identified patterns of polypharmacy are analysed in detail below. 

### Cardiovascular pattern

The cardiovascular pattern is present in men and women from age 45 years onwards (Table **3**). Among middle-aged women (i.e., 45-64 years), the pattern has a prevalence of 4% and comprises antidiabetics, lipid-lowering drugs, diuretics, angiotensin-converting enzyme (ACE) inhibitors, angiotensin II receptor antagonists (ARBs), beta-blockers, calcium channel blockers, antiplatelet agents, and antiglaucoma eye drops. Except for the latter, the other drugs are indicated for the treatment of cardiovascular risk factors (i.e., hypertension, diabetes mellitus, and dyslipidemia) [12] and for the primary or secondary prevention of clinically established arteriosclerosis [13]. Among the reasons for the presence of antiglaucoma medications in this pattern, it may be noted that both hypertension [14] and diabetes [15] are risk factors for glaucoma and that glaucoma may be caused by one of the drugs included in the pattern (i.e., diuretics) [16]. In the latter case, this would be an example of treatment of a side effect that is not included in the main CPGs for cardiovascular disease. 

**Table 3:** Cardiovascular pattern composition by age and sex.

	**15-44 years**	**45-64 years**	**≥65 years**
**Women**		Ace inhibitors, plain	Ace inhibitors, plain
		Angiotensin ii antagonists, combinations	Antigout preparations
		Angiotensin ii antagonists, plain	Antithrombotic agents
		Antiglaucoma preparations and miotics	Beta blocking agents
		Antithrombotic agents	Blood glucose lowering drugs, excluding insulins
		Beta blocking agents	Cardiac glycosides
		Blood glucose lowering drugs, excluding insulins	High-ceiling diuretics
		High-ceiling diuretics	Insulins and analogues
		Insulins and analogues	Potassium-sparing agents
		Lipid modifying agents, plain	Selective calcium channel blockers with direct cardiac effects
		Selective calcium channel blockers with mainly vascular effects	Vasodilators used for cardiac diseases
**Men**		Ace inhibitors, plain	Ace inhibitors, plain
		Angiotensin ii antagonists, combinations	Antithrombotic agents
		Angiotensin ii antagonists, plain	Beta blocking agents
		Antigout preparations	Drugs for peptic ulcer and gastro-oesophageal reflux disease
		Antithrombotic agents	High-ceiling diuretics
		Beta blocking agents	Insulins and analogues
		Blood glucose-lowering drugs, excluding insulins	Lipid modifying agents, plain
		Drugs for peptic ulcer and gastro-oesophageal reflux disease	Potassium-sparing agents
		High-ceiling diuretics	Selective calcium channel blockers with direct cardiac effects
		Insulins and analogues	Selective calcium channel blockers with mainly vascular effects
		Lipid modifying agents, plain	Vasodilators used in cardiac diseases
		Selective calcium channel blockers with mainly vascular effects	
		Vasodilators used in cardiac diseases	

After 65 years of age, the prevalence of this pattern increases in women, being present in almost one out of ten. In addition, new drugs are added as possible treatments for some of the complications in these patients, such as nitrites for ischaemic heart disease, digoxin and aldosterone inhibitors for heart failure, and hypouricemic drugs, which are frequently prescribed for cardiovascular disease [17] as a possible treatment of the side-effects of diuretics. It is noteworthy that, in this age group, there are no ARBs, lipid-lowering drugs or antiglaucoma medications that are used. The use of ARBs both as monotherapy [18] or in combination with (ACE) inhibitors [19] has been questioned in last years. Regarding lipid-lowering drugs, a recent meta-analysis revealed no reduction in the overall mortality of women treated with statins for secondary prevention [20]. As for antiglaucoma medication, no explanation could be found for its disappearance from the cardiovascular pattern. Finally, the absence of proton pump inhibitors (PPIs) in this pattern is striking; the use of these drugs as gastroprotectants is widely recommended for patients taking antiplatelet agents [21].

In men who are 45 to 64 years of age, the pattern almost triples in prevalence compared with women (11%) and is very similar in terms of its composition. This greater prevalence of the pattern is consistent with the increased frequency of cardiovascular disease in men. Other relevant differences with regard to women are as follows: nitrites are used in ischaemic heart disease, which occurs earlier in men; hypouricemic drugs begin to be used; and PPIs appear, which could be associated with the use of antiplatelet agents in patients for whom these drugs are indicated in the CPGs [21]. 

During old age, after 65 years, the prevalence of this pattern of polypharmacy increases until it is present in one out of four men. Regarding the therapeutic groups that comprise this pattern, there are three important differences compared with women of the same age: 1) the sustained use of PPIs, 2) the absence of drugs for heart failure, and 3) the additional use of lipid-lowering drugs. The latter difference may be due to an increased cardiovascular risk in men and their increased incidence of ischaemic heart disease and cerebrovascular disease—with the effectiveness of lipid-lowering drugs being less evident in women [20]. It is not possible to rule out the possible underdiagnosis and undertreatment of this disease in women, as has been previously described [22].

In summary, this study allowed for the identification of a clinically consistent cardiovascular pattern of polypharmacy that exhibits a clear relation to the recently described cardio-metabolic multimorbidity pattern (2) and that develops differently based on the gender and age. The present investigation also revealed the presence of unexpected associations (i.e., antiglaucoma drugs in middle-aged women) or the absence of certain drugs (such as PPIs) in older women, which opens new and relevant research areas. 

### Depression-anxiety pattern

This is a highly prevalent pattern, present in all age and gender groups, with a wide range of variation in their composition and frequency of occurrence (i.e., at advanced ages, the frequency ranges between 37% in women and 0.3% in men) (Table **4**). The lower prevalence of this pattern in men is striking, a difference that would presumably diminish if certain drugs that had values slightly below the established cut-off were considered (e.g., anxiolytics with a factor score of 0.23). The group of drugs that comprise this pattern (i.e., antidepressants, antipsychotics, anxiolytics, and sedatives) are used in clinical practice for the treatment of anxiety disorders, depression, and neurosis. The presence of antiepileptic drugs such as gabapentin, both in men and women under 65 years of age, is remarkable. This drug might be used off-label as a modulator of behaviour disorders in psychotic patients [23,24]; the drug might also be administered as an adjuvant medication for pain uncontrollable with regular analgesia.

**Table 4:** Depression-anxiety pattern composition according to age and sex.

	**15-44 years**	**45-64 years**	**≥65 years**
**Women**	Antiandrogens	Antacids	Antidepressants
	Antidepressants	Antidepressants	Antiepileptics
	Antiepileptics	Antiepileptics	Antiinflammatory and antirheumatic products, non-steroids
	Antipsychotics	Antiinflammatory and antirheumatic products, non-steroids	Anxiolytics
	Anxiolytics	Antipsychotics	Calcium
	Muscle relaxants, centrally acting agents	Anxiolytics	Drugs for peptic ulcer and gastro-oesophageal reflux disease
		Drugs for peptic ulcer and gastro-oesophageal reflux disease	Iron preparations
		Hypnotics and sedatives	Laxatives
		Laxatives	Opioids
		Muscle relaxants, centrally acting agents	Other urologicals, including antispasmodics
		Opioids	Other analgesics and antipyretics
		Propulsives	Propulsives
			Psychostimulants, agents used for ADHD and nootropics
			Topical products for joint and muscular pain
			Vitamin B12 and folic acid
**Men**	Antidepressants	Antacids	Agents for treatment of haemorrhoids and anal fissures for topical use
	Antiepileptics	Antidepressants	Antidepressants
	Antipsychotics	Antiepileptics	Antipsychotics
	Anxiolytics	Antipsychotics	Capillary stabilising agents
	Drugs for peptic ulcer and gastro-oesophageal reflux disease	Anxiolytics	Cardiac glycosides
	Lipid modifying agents, plain	Hypnotics and sedatives	Dopaminergic agents
		Laxatives	Laxatives
		Opioids	
		Propulsives	

Despite the clinical coherence in most of the associations between drugs, other associations were detected, for which it has not been possible to find a causal explanation. This is the case of antiandrogens (i.e., ethinyloestradiol and cyproterone) in young women aged 15 to 44 years, although future studies should continue to shed light on the possible association between acne beginning at puberty and depression [25].

In young men aged 15 to 44 years, lipid-lowering medications and drugs for peptic ulcer and gastro-oesophageal reflux are added to the pattern. The presence of the latter medications could be justified because of the association between antidepressants and upper gastrointestinal tract bleeding [26] or gastro-oesophageal reflux disease [27]. However, it might be asked why gastroprotectants are not part of this pattern in women of this age group, which is certainly a question for future research. 

Among patients aged 45 to 64 years, opioids are added to this pattern, which may be explained by the association for depression-somatisation pain [28,29]. In women, anti-inflammatory drugs, antirheumatic drugs, and centrally acting muscle relaxants are also included. The presence of gastroprotectants (i.e., gastroprokinetic agents, laxatives, and drugs for peptic ulcer and gastroesophageal reflux) could be due to the treatment of side effects of antidepressants. 

After 65 years of age, this pattern of polypharmacy corresponds to a clinical situation of serious mental illness and it includes antidepressants, anxiolytics, and anticonvulsants in women as well as antidepressants, antipsychotics, and dopaminergic agents in men. Furthermore, in women, an analgesic component appears in this pattern, which consists of the same drug groups as that for middle-aged women (e.g., opioids) and topical preparations for joint pain. The use of laxatives and gastroprokinetic agents is present in both sexes, most likely in response to the adverse effects of opioids [30]. In women, this pattern may reflect the treatment of fibromyalgia syndrome [31], although they are also administered calcium, iron, and vitamin B12 as possible preventive treatments or in the case of overt deficits. 

In men over 65 years of age, it is possible to hypothesise that this pattern relates to the treatment of psychogeriatric diseases, comprising dopaminergic agents prescribed for parkinsonism, in addition to the previously mentioned drugs [32]. In the latter group, no acceptable explanations have been found for the presence of cardiac glycosides, capillary agents (i.e., diosmin and oxerutins), and/or topical antihaemorrhoidal agents.

In conclusion, some of the findings within this complex pattern indicate associations between drugs that are difficult to explain from the available clinical knowledge. It has also been impossible to elucidate the causes of certain differences observed between the sexes, such as the absence of antipsychotics in older women or the absence of vitamin B12 and folic acid in older men.

### Acute respiratory infection (ARI) pattern

This pattern is present in men from 15 to 44 years of age and in women up to 64 years (Table **5**). In women aged 15 to 44 years, the prevalence of the ARI pattern is high (8%) and comprises many medications related to the treatment of ARI and its complications (i.e., residual cough or bronchospasm). We found two associations that could be related to treatment with other drugs: PPIs, which are gastroprotectants used in patients treated with NSAIDs, and antifungal drugs for the treatment of candidiasis secondary to antibiotics. Moreover, this pattern includes two groups of drugs for which we found no clinically reasonable explanation: topical corticosteroids and anxiolytics. 

**Table 5:** ARI pattern composition by age and sex.

	**15-44 years**	**45-64 years**	**≥65 years**
**Women**	Adrenergics, inhalants	Adrenergics, inhalants	
	Antifungals for topical use	Antihistamines for systemic use	
	Antihistamines for systemic use	Antiinfectives for systemic use	
	Antiinfectives and antiseptics, excluding combinations with corticosteroids	Corticosteroids for systemic use, plain	
	Antiinfectives for systemic use	Cough suppressants, excluding combinations with expectorants	
	Antiinflammatory and antirheumatic products, non-steroids	Decongestants and other nasal preparations for topical use	
	Anxiolytics	Expectorants, excluding combinations with cough suppressants	
	Corticosteroids, plain	Nasal decongestants for systemic use	
	Cough suppressants, excluding combinations with expectorants	Other analgesics and antipyretics	
	Decongestants and other nasal preparations for topical use		
	Drugs for peptic ulcer and gastro-oesophageal reflux disease		
	Expectorants, excluding combinations with cough suppressants		
	Opioids		
	Other analgesics and antipyretics		
**Men**	Antiinfectives for systemic use		
	Antiinflammatory and antirheumatic products, non-steroids		
	Cough suppressants, excluding combinations with expectorants		
	Drugs for peptic ulcer and gastro-oesophageal reflux disease		
	Expectorants, excluding combinations with cough suppressants		
	Other analgesics and antipyretics		

In middle-aged women, the prevalence of this pattern is very similar to that in young women (7%), but with interesting therapeutic absences: 1) NSAIDs, perhaps due to underprescribing because of the risk of digestive and cardiovascular side effects (consequently, PPIs also do not appear); 2) antifungals for topical use, most likely due to the lower incidence of vaginal candidiasis in postmenopausal women [33]; and 3) the two treatment groups of difficult clinical explanation found among younger women (i.e., topical corticosteroids and anxiolytics). Furthermore, the association of systemic corticosteroids, due to the worsening of COPD, is noteworthy [34].

Between 15 and 44 years of age, the prevalence of this pattern in men is one-half that in women of the same age (4%) and comprises analgesics, NSAIDs with their associated PPIs, antibiotics, and drugs for the symptomatic treatment of cough. With respect to women of the same age, opioids, sympathomimetic bronchodilators, nasal decongestants, and the previously discussed therapeutic groups of difficult clinical explanation disappear.

In summary, the ARI pattern is highly prevalent—probably due to the use of pharmacy data from the months of January and February—and comprises a group of drugs administered for the same category of diseases, including medications (e.g., vaginal antifungal agents and PPIs) that are used to treat complications of these illnesses or the side effects of other drugs. Some of the associations that have been found, especially in young women, should be further investigated.

### COPD pattern

The COPD pattern appears in men over 44 years of age, and it is delayed in women until the age of 65 years (Table **6**). This pattern includes drugs for the treatment of airway obstruction (i.e., systemic corticosteroids, expectorants, and antihistamines) along with other medications for the treatment of symptoms (e.g., analgesics, antitussives, and antipyretics). The presence of antihistamines and antitussives indicates the differential diagnosis between COPD and other allergic and/or asthmatic processes, in which the presence of cough and wheezing is common. As a pattern of polypharmacy that corresponds to a chronic and advanced disease, the fact that this pattern appears in a more delayed form in women may reflect a lower prevalence and intensity of smoking in women [35]. 

**Table 6:** COPD pattern composition by age and sex.

	**15-44 years**	**45-64 years**	**≥65 years**
**Women**			Adrenergics, inhalants
			Antihistamines for systemic use
			Antiinfectives
			Antiinfectives for systemic use
			Antiinfectives for systemic use
			Antiinflammatory agents and antiinfectives in combination
			Corticosteroids for systemic use, plain
			Cough suppressants, excluding combinations with expectorants
			Decongestants and other nasal preparations for topical use
			Expectorants, excluding combinations with cough suppressants
			Other drugs for obstructive airway diseases, inhalants
**Men**		Adrenergics, inhalants	Adrenergics, inhalants
		Antihistamines for systemic use	Antihistamines for systemic use
		Antiinfectives for systemic use	Antiinfectives
		Corticosteroids for systemic use, plain	Antiinfectives for systemic use
		Cough suppressants, excluding combinations with expectorants	Antiinflammatory agents and antiinfectives in combination
		Decongestants and other nasal preparations for topical use	Antiinflammatory and antirheumatic products, non-steroids
		Expectorants, excluding combinations with cough suppressants	Antiinflammatory agents
		Other analgesics and antipyretics	Calcium
		Other drugs for obstructive airway diseases, inhalants	Corticosteroids for systemic use, plain
			Cough suppressants, excluding combinations with expectorants
			Drugs for peptic ulcer and gastro-oesophageal reflux disease
			Expectorants, excluding combinations with cough suppressants
			Opioids
			Other drugs for obstructive airway diseases, inhalants
			Other analgesics and antipyretics
			Other ophthalmologicals

In men over 64 years of age, this pattern is present in one out of four individuals and comprises the same drugs that are used by middle-aged individuals, to which NSAIDs and drugs for peptic ulcer and gastro-oesophageal reflux are added. The presence of calcium in this pattern is possibly due to its use for the prevention of osteoporosis in patients taking corticosteroids. Opioids also appear, prescribed probably as antitussives or analgesics in patients with COPD [36]. 

In older women, this pattern has a prevalence of 7% and exhibits certain peculiarities, namely, the absence of opioids, eye lubricants, and calcium. It is necessary to consider what the possible causes are. Finally, it should be mentioned that PPIs, which are prescribed for protection against NSAIDs or the gastric reflux induced by beta-2 mimetics, do not appear to be associated to this pattern among women.

### Rhinitis-asthma pattern

The rhinitis-asthma pattern occurs with a low frequency and exclusively in young men (0.1%), and it is highly consistent from the clinical viewpoint (Table **7**). This pattern comprises three therapeutic groups used for two pathologies with a common aetiology: nasal corticosteroids and systemic antihistamines for the treatment of allergic rhinitis [37] and inhaled beta-adrenergic agonists for the treatment of asthma [38]. 

**Table 7:** Rhinitis-asthma pattern composition according to age and sex.

	**15-44 years**	**45-64 years**	**≥65 years**
**Women**			
**Men**	Adrenergics, inhalants		
	Antihistamines for systemic use		
	Decongestants and other nasal preparations for topical use		

### Pain pattern

The pain pattern appears only in men who are 45 to 64 years of age, affecting 5% of the patients in this group (Table **8**). This pattern includes analgesic drugs (e.g., acetaminophen, and opioids), NSAIDs, central muscle relaxants, and other medications for the treatment of side effects (i.e., drugs for peptic ulcer). The presence of low-ceiling diuretics (e.g., chlorthalidone and indapamide) that are used in clinical practice for mild essential hypertension is prominent [39]. It is possible that these drugs are prescribed to combat the hypertensive effect of NSAIDs. This finding would be clinically relevant if the physician were addressing the potential gastrolesivity of NSAIDs but not the possible vascular-renal damage. If future longitudinal studies confirm this finding, then specific indications in the CPGs should be designed to recommend against the prescription of NSAIDs as long-term analgesics.

**Table 8:** Pain pattern composition according to age and sex.

	**15-44 years**	**45-64 years**	**≥65 years**
**Women**			
**Men**		Antiinflammatory and antirheumatic products, non-steroids	
		Cough suppressants, excluding combinations with expectorants	
		Drugs for peptic ulcer and gastro-oesophageal reflux disease	
		Low-ceiling diuretics, excluding thiazides	
		Muscle relaxants, centrally acting agents	
		Opioids	
		Other analgesics and antipyretics	
		Topical products for joint and muscular pain	

### Menopause pattern

The menopause pattern has a low prevalence—1% of women aged 45-64 years-(Table **9**) and includes two groups of drugs: the first group, consisting of oestrogen, bisphosphonates, and calcium for the prevention or treatment of the effects of menopause (i.e., hot flashes and osteoporosis), and the second group, consisting of drugs prescribed for the symptomatic relief of some of the side-effects described in the literature (e.g., ocular inflammation caused by bisphosphonates) [40]. Despite its low prevalence, the existence of this pattern is striking, to the extent that various studies have demonstrated that both oestrogen and bisphosphonates have potentially serious side effects [41].

**Table 9:** Menopause pattern composition according to age and sex.

	**15-44 years**	**45-64 years**	**≥65 years**
**Women**		Calcium	
		Decongestants and antiallergics	
		Drugs affecting bone structure and mineralisation	
		Oestrogens	
		Other ophthalmologicals	
**Men**			

### Strengths and limitations

The main strengths of the present study are the large population size and the quality of the data in the pharmacy database (i.e., pharmacy billing records), which yield greater reliability and representativeness compared with other studies that are based on medical records or surveys of drug use [42].

The exploratory factor analysis that was employed in this study, in addition to being the technique that best responds to the proposed objective, was performed according to the recommendations of Costello et al. [43]. Moreover, the goodness-of-fit values of the models expressed as the cumulative percentage of variance (i.e., between 19.47% and 58.54%) and the sampling adequacy (i.e., a KMO parameter between 0.66 and 0.73) are above the acceptable limits.

Although several hypotheses have been presented regarding the pathophysiological processes that underlie the seven polypharmacy patterns revealed in this study, the former must be interpreted with the necessary caution since the study design (i.e., transversal) does not allow for the establishment of the sequence in which medications cluster within a pattern. Longitudinal studies would be necessary to corroborate the suggested causal associations and to elucidate those associations that could not be explained in the present study.

Another limitation of this study stems from the lack of information on the actual use of drugs by the patients because the pharmacy database contains information on the drugs that are prescribed and dispensed at the pharmacy, without providing proof of their consumption. Yet, this is currently the data source of choice when carrying out population-based studies on drug use, as stated previously. 

Access to data on the use of over-the-counter (OTC) medications was not available, which could lead to an underestimation of the actual drug utilisation. Additionally, only patients seen by their GP in 2008 were included in the study. By doing so, prevalence figures of polypharmacy patterns may overestimate the real frequency in the studied age and sex groups. However, we believe this drawback may not have interfered with the essential objective of the study, that is, to analyse systematic associations in drug prescription and use.

Because these data were obtained from seven different health centres, there may be differences in the socioeconomic status and/or accessibility to public health care among members of the target population; however, this heterogeneity across individuals would increase the external validity of the results. Some variability might also exist between the centres in the drug prescription practices of their health professionals, which would directly influence the results. Similarly, potential differences in the organisational or functional aspects of each centre (e.g., organisation of the pharmacy or laboratory) could indirectly influence the associations obtained. Therefore, the fact that the therapeutic group variable has been analysed with the information obtained up to the third level of the ATC classification (i.e., by the therapeutic family rather than by the active ingredient) would reduce the possible variability between the centres and professionals in determining the patterns of polypharmacy.

No distinction was made regarding the level of care where prescriptions originated albeit the importance of induced prescription in primary care, as stated in the introduction. Future studies should consider this information in order to elucidate the impact of the primary-secondary care interface on the nature and intensity of polypharmacy. 

### Comparisons with other studies

Comparing our results with those of other studies is difficult, mainly because of the lack of similar studies in the scientific literature. In fact, the present study is the first investigation of the patterns of polypharmacy to be conducted in the general population. Research in the field of pharmacoepidemiology remains driven by the hermetic single-disease-single-medication framework required in clinical trials [44]. However, in no cases have combinations of medications been broadly analysed at a population level to determine the specific interactions and synergistic effects between them. Several studies have examined the co-prescription of drugs, but only for index-specific diseases. For example, Hoffmann et al. [45] described comorbidity and polypharmacy in patients with dementia, as did Franssen et al. [46] for patients with COPD.

## Conclusions

This study revealed systematic associations in drug prescription that affect a significant proportion of the population and that are present in all of the age and sex groups that were studied. These associations yield patterns of polypharmacy that are consistent from the pharmacological and clinical points of view. Seven patterns of polypharmacy were identified: cardiovascular, depression-anxiety, acute respiratory infection (ARI), chronic obstructive pulmonary disease (COPD), rhinitis-asthma, pain, and menopause.

The clinical interpretation of the relations between the drugs that give rise to these patterns allows us to hypothesise the existence of underlying causal factors that are often related, not to the disease itself, but to the adverse effects of the prescribed treatments, which would explain many of the observed associations. 

The present study highlights the necessity of developing future longitudinal studies, including the joint analysis of diseases and drugs, which would facilitate the validation of certain potential interactions described in this article. This information would be an essential source for the development of CPGs addressing multimorbidity and the design of clinical management strategies and models of care with the capacity to respond appropriately to the real health needs of patients with multiple chronic conditions.

## Supporting Information

File S1 **This file contains Figure A.** Figure A, Scree plots for the different age and sex groups.
(DOC)

File S2 **This file contains Table A – Table F.** Table A, Factor scores for women between 15 and 44 years of age. Table B, Factor scores for women between 45 and 64 years of age. Table C, Factor scores for women over 64 years of age. Table D, Factor scores for men between 15 and 44 years of age. Table E, Factor scores for men between 45 and 64 years of age. Table F, Factor scores for men over 64 years of age.
(DOC)
