# Supplementary material for: Polypharmacy Patterns: Unravelling Systematic Associations between Prescribed Medications
Source: PLoS One. 2013 Dec 20;8(12):e84967. doi: 10.1371/journal.pone.0084967 (PMC3869920; doi:10.1371/journal.pone.0084967)
Supplement: File S1 — This file contains Figure A. Figure A, Scree plots for the different age and sex groups. (DOC) [file pone.0084967.s001.doc]

Supplemental file S1

Figure A - Scree plots for the different age and sex groups
