# Supplementary material for: Polypharmacy Patterns: Unravelling Systematic Associations between Prescribed Medications
Source: PLoS One. 2013 Dec 20;8(12):e84967. doi: 10.1371/journal.pone.0084967 (PMC3869920; doi:10.1371/journal.pone.0084967)
Supplement: File S2 — This file contains Table A – Table F. Table A, Factor scores for women between 15 and 44 years of age. Table B, Factor scores for women between 45 and 64 years of age. Table C, Factor scores for women over 64 years of age. Table D, Factor scores for men between 15 and 44 years of age. Table E, Factor scores for men between 45 and 64 years of age. Table F, Factor scores for men over 64 years of age. (DOC) [file pone.0084967.s002.doc]

Supplemental file S2

Table A - Factor scores for women between 15 and 44 years of age

| **ATC** | **Drug** | **Factor1** | **Factor2** |
| --- | --- | --- | --- |
| **J** | **Antiinfectives for systemic use** | **0.71** | -0.09 |
| **N02B** | **Other analgesics and antipyretics** | **0.66** | -0.12 |
| **R05C** | **Expectorants, excluding combinations with cough suppressants** | **0.61** | 0.01 |
| **M01A** | **Antiinflammatory and antirheumatic products, non-steroids** | **0.55** | 0.20 |
| **R01A** | **Decongestants and other nasal preparations for topical use** | **0.50** | -0.04 |
| **R06A** | **Antihistamines for systemic use** | **0.48** | 0.00 |
| **R05D** | **Cough suppressants, excluding combinations with expectorants** | **0.47** | 0.02 |
| **A02B** | **Drugs for peptic ulcer and gastro-oesophageal reflux disease** | **0.46** | 0.25 |
| **R03A** | **Adrenergics, inhalants** | **0.45** | 0.06 |
| **G01A** | **Antiinfectives and antiseptics, excluding combinations with corticosteroids** | **0.39** | -.0.19 |
| **D07A** | **Corticosteroids, plain** | **0.35** | 0.02 |
| **N02A** | **Opioids** | **0.34** | 0.30 |
| **D01A** | **Antifungals for topical use** | **0.32** | -0.09 |
| **N05B** | **Anxiolytics** | **0.35** | **0.41** |
| **N06A** | **Antidepressants** | 0.18 | **0.68** |
| **N03A** | **Antiepileptics** | 0.26 | **0.43** |
| **M03B** | **Muscle relaxants, centrally acting agents** | 0.20 | **0.43** |
| **G03H** | **Antiandrogens** | 0.00 | **0.38** |
| **N05A** | **Antipsychotics** | 0.18 | **0.36** |
| A03F | Propulsives | 0.28 | 0.08 |
| H03A | Thyroid preparations | 0.29 | 0.06 |
| B03B | Vitamin B12 and folic acid | 0.24 | -.0.35 |
| B03A | Iron preparations | 0.21 | -0.37 |
| H03C | Iodine therapy | 0.18 | -0.91 |

**KMO: 0.66**

**% cumulative variance: 32.36%**

**Table B - Factor scores for women between 45 and 64 years of age**

| **ATC** | **Drug** | **Factor1** | **Factor2** | **Factor3** | **Factor4** |
| --- | --- | --- | --- | --- | --- |
| **A10B** | **Blood glucose lowering drugs, excluding insulins** | **0.77** | -0.10 | -0.01 | -0.11 |
| **A10A** | **Insulins and analogues** | **0.76** | -0.05 | 0.11 | -0.27 |
| **C10A** | **Lipid modifying agents, plain** | **0.70** | 0.04 | -0.03 | 0.05 |
| **B01A** | **Antithrombotic agents** | **0.68** | 0.07 | -0.02 | -0.04 |
| **C08C** | **Selective calcium channel blockers with mainly vascular effects** | **0.61** | 0.04 | -0.02 | 0.03 |
| **C07A** | **Beta blocking agents** | **0.56** | 0.03 | -0.16 | 0.06 |
| **C03C** | **High-ceiling diuretics** | **0.50** | 0.16 | 0.02 | -0.08 |
| **C09A** | **ACE inhibitors, plain** | **0.47** | -0.04 | -0.02 | -0.09 |
| **C09C** | **Angiotensin II antagonists, plain** | **0.46** | -0.06 | 0.05 | 0.02 |
| **C09D** | **Angiotensin II antagonists, combinations** | **0.38** | 0.03 | 0.05 | 0.01 |
| **S01E** | **Antiglaucoma preparations and miotics** | **0.32** | -0.06 | 0.06 | 0.15 |
| **N06A** | **Antidepressants** | 0.04 | **0.66** | -0.09 | -0.07 |
| **N03A** | **Antiepileptics** | 0.04 | **0.61** | -0.08 | -0.02 |
| **N05B** | **Anxiolytics** | 0.11 | **0.57** | -0.04 | -0.02 |
| **A02B** | **Drugs for peptic ulcer and gastro-oesophageal reflux disease** | 0.25 | **0.55** | 0.15 | 0.10 |
| **N02A** | **Opioids** | 0.05 | **0.50** | 0.12 | 0.10 |
| **A03F** | **Propulsives** | -0.05 | **0.47** | 0.10 | -0.03 |
| **A06A** | **Laxatives** | 0.00 | **0.47** | -0.02 | 0.11 |
| **N05C** | **Hypnotics and sedatives** | 0.05 | **0.47** | -0.01 | 0.00 |
| **N05A** | **Antipsychotics** | -0.02 | **0.47** | -0.02 | -0.07 |
| **A02A** | **Antacids** | 0.10 | **0.44** | 0.12 | -0.37 |
| **M01A** | **Antiinflammatory and antirheumatic products, non-steroids** | 0.01 | **0.38** | 0.24 | 0.20 |
| **M03B** | **Muscle relaxants, centrally acting agents** | -0.06 | **0.34** | 0.04 | 0.09 |
| **J** | **Antiinfectives for systemic use** | -0.02 | 0.07 | **0.77** | -0.14 |
| **R05C** | **Expectorants, excluding combinations with cough suppressants** | -0.02 | -0.02 | **0.75** | -0.10 |
| **R03A** | **Adrenergics, inhalants** | 0.02 | 0.02 | **0.60** | -0.05 |
| **R01A** | **Decongestants and other nasal preparations for topical use** | -0.09 | -0.01 | **0.59** | 0.06 |
| **R05D** | **Cough suppressants, excluding combinations with expectorants** | 0.02 | -0.02 | **0.55** | -0.03 |
| **R06A** | **Antihistamines for systemic use** | -0.02 | -0.06 | **0.51** | 0.10 |
| **R01B** | **Nasal decongestants for systemic use** | 0.03 | -0.08 | **0.49** | 0.07 |
| **N02B** | **Other analgesics and antipyretics** | 0.10 | 0.25 | **0.46** | 0.03 |
| **H02A** | **Corticosteroids for systemic use, plain** | 0.00 | 0.14 | **0.44** | 0.06 |
| **A12A** | **Calcium** | 0.00 | 0.14 | -0.03 | **0.64** |
| **S01G** | **Decongestants and antiallergics** | -..02 | -0.16 | 0.17 | **0.51** |
| **M05B** | **Drugs affecting bone structure and mineralisation** | .0.07 | 0.12 | -0.02 | **0.48** |
| **S01X** | **Other ophthalmologicals** | 0.06 | 0.14 | 0.14 | **0.32** |
| **G03C** | **Oestrogens** | 0.00 | 0.12 | 0.03 | **0.31** |
| S01A | Antiinfectives | 0.12 | -0.08 | 0.14 | 0.27 |
| G03X | Other sex hormones and modulators of the genital system | -0.03 | 0.04 | -0.02 | 0.26 |
| C05C | Capillary stabilising agents | 0.17 | 0.20 | 0.05 | 0.17 |
| M02A | Topical products for joint and muscular pain | 0.08 | 0.26 | 0.14 | 0.16 |
| D01A | Antifungals for topical use | 0.03 | 0.19 | 0.15 | 0.16 |
| C03B | Low-ceiling diuretics, excluding thiazides | 0.25 | -0.02 | -0.01 | 0.16 |
| H03A | Thyroid preparations | 0.16 | 0.09 | 0.01 | 0.13 |
| N02C | Antimigraine preparations | -0.09 | 0.29 | -0.06 | 0.10 |
| D07A | Corticosteroids, plain | 0.03 | 0.26 | 0.08 | 0.06 |
| N07C | Antivertigo preparations | 0.01 | 0.26 | 0.01 | 0.02 |
| C09B | ACE inhibitors, combinations | 0.27 | 0.04 | 0.00 | 0.01 |
| B03A | Iron preparations | 0.07 | 0.21 | 0.08 | -0.14 |

**KMO: 0.72**

**% cumulative variance: 33.11%**

**Table C - Factor scores for women over 64 years of age**

| | **ATC** | **Drug** | **Factor1** | **Factor2** | **Factor3** | | --- | --- | --- | --- | --- | | **A02B** | **Drugs for peptic ulcer and gastro-oesophageal reflux disease** | **0.61** | 0.11 | 0.23 | | **A06A** | **Laxatives** | **0.44** | 0.01 | 0.11 | | **N02A** | **Opioids** | **0.43** | 0.04 | 0.04 | | **N05B** | **Anxiolytics** | **0.41** | 0.01 | 0.06 | | **N06A** | **Antidepressants** | **0.40** | -0.04 | 0.06 | | **A03F** | **Propulsives** | **0.37** | 0.06 | 0.01 | | **M01A** | **Antiinflammatory and antirheumatic products, non-steroids** | **0.37** | 0.13 | -0.21 | | **B03A** | **Iron preparations** | **0.36** | -0.10 | 0.24 | | **N02B** | **Other analgesics and antipyretics** | **0.36** | 0.28 | 0.08 | | **M02A** | **Topical products for joint and muscular pain** | **0.34** | 0.11 | 0.01 | | **N03A** | **Antiepileptics** | **0.34** | 0.01 | 0.11 | | **A12A** | **Calcium** | **0.34** | 0.04 | -0.26 | | **N06B** | **Psychostimulants, agents used for ADHD and nootropics** | **0.32** | -0.03 | 0.03 | | **G04B** | **Other urologicals, including antispasmodics** | **0.32** | -0.10 | -0.09 | | **B03B** | **Vitamin B12 and folic acid** | **0.30** | -0.09 | 0.17 | | **J** | **Antiinfectives for systemic use** | 0.04 | **0.66** | 0.08 | | **R03A** | **Adrenergics, inhalants** | -0.12 | **0.66** | 0.19 | | **R05C** | **Expectorants, excluding combinations with cough suppressants** | 0.05 | **0.58** | 0.06 | | **R03B** | **Other drugs for obstructive airway diseases, inhalants** | -0.04 | **0.54** | 0.18 | | **H02A** | **Corticosteroids for systemic use, plain** | -0.01 | **0.53** | 0.11 | | **R06A** | **Antihistamines for systemic use** | 0.05 | **0.51** | 0.08 | | **R01A** | **Decongestants and other nasal preparations for topical use** | -0.05 | **0.48** | -0.20 | | **R05D** | **Cough suppressants, excluding combinations with expectorants** | 0.05 | **0.38** | 0.02 | | **S01C** | **Antiinflammatory agents and antiinfectives in combination** | 0.04 | **0.34** | -0.04 | | **S01A** | **Antiinfectives** | 0.16 | **0.31** | -0.09 | | **B01A** | **Antithrombotic agents** | 0.19 | 0.01 | 0.67 | | **C03C** | **High-ceiling diuretics** | 0.10 | 0.13 | 0.60 | | **C01A** | **Cardiac glycosides** | -0.09 | 0.02 | 0.56 | | **C03D** | **Potassium-sparing agents** | -0.11 | 0.06 | 0.56 | | **C01D** | **Vasodilators used in cardiac diseases** | 0.01 | 0.17 | 0.55 | | **A10A** | **Insulins and analogues** | 0.07 | -0.01 | 0.53 | | **C07A** | **Beta blocking agents** | 0.08 | 0.00 | 0.41 | | **M04A** | **Antigout preparations** | 0.11 | -0.23 | 0.40 | | **C08D** | **Selective calcium channel blockers with direct cardiac effects** | -0.09 | 0.22 | 0.38 | | **A10B** | **Blood glucose-lowering drugs, excluding insulins** | 0.05 | -0.05 | 0.33 | | **C09A** | **ACE inhibitors, plain** | 0.01 | 0.02 | 0.33 | | C10A | Lipid modifying agents, plain | 0.15 | -0.03 | 0.29 | | C09C | Angiotensin II antagonists, plain | 0.06 | 0.06 | 0.21 | | C08C | Selective calcium channel blockers with mainly vascular effects | 0.19 | -0.05 | 0.21 | | C01E | Other cardiac preparations | 0.16 | 0.02 | 0.12 | | S01E | Antiglaucoma preparations and miotics | 0.08 | 0.13 | 0.11 | | N05A | Antipsychotics | 0.28 | -0.08 | 0.11 | | N05C | Hypnotics and sedatives | 0.28 | 0.05 | 0.08 | | C09D | Angiotensin II antagonists, combinations | 0.10 | 0.05 | 0.07 | | H03A | Thyroid preparations | 0.11 | 0.05 | 0.07 | | N06D | Anti-dementia drugs | 0.24 | -0.24 | 0.06 | | D07A | Corticosteroids, plain | 0.18 | 0.18 | 0.03 | | D01A | Antifungals for topical use | 0.27 | 0.13 | 0.03 | | C09B | Ace inhibitors, combinations | 0.01 | -0.03 | 0.03 | | C05C | Capillary stabilising agents | 0.27 | 0.03 | 0.02 | | C03E | Diuretics and potassium-sparing agents in combination | 0.03 | 0.02 | 0.01 | | C04A | Peripheral vasodilators | 0.22 | -0.05 | 0.01 | | C05A | Agents for treatment of haemorrhoids and anal fissures for topical use | 0.15 | 0.22 | 0.00 | | S01B | Antiinflammatory agents | 0.11 | 0.25 | -0.02 | | N07C | Antivertigo preparations | 0.21 | -0.01 | -0.06 | | C03B | Low-ceiling diuretics, excluding thiazides | 0.07 | -0.04 | -0.07 | | N04B | Dopaminergic agents | 0.24 | -0.24 | -0.08 | | A02A | Antacids | 0.20 | 0.12 | -0.08 | | S01G | Decongestants and antiallergics | 0.18 | 0.22 | -0.08 | | S01X | Other ophthalmologicals | 0.29 | 0.15 | -0.08 | | M03B | Muscle relaxants, centrally acting agents | 0.21 | 0.12 | -0.10 | | C03A | Low-ceiling diuretics, thiazides | -0.05 | 0.06 | -0.10 | | D06A | Antibiotics for topical use | 0.19 | 0.12 | -0.13 | | A01A | Stomatological preparations | 0.29 | 0.22 | -0.13 | | G03C | Oestrogens | 0.16 | 0.12 | -0.16 | | S02C | Corticosteroids and antiinfectives in combination (otologicals) | 0.09 | 0.24 | -0.18 | | M05B | Drugs affecting bone structure and mineralisation | 0.28 | 0.09 | -0.18 | | G03X | Other sex hormones and modulators of the genital system | 0.11 | 0.00 | -0.36 | |
| --- | --- | --- | --- | --- | --- | --- | --- | --- | --- | --- | --- | --- | --- | --- | --- | --- | --- | --- | --- | --- | --- | --- | --- | --- | --- | --- | --- | --- | --- | --- | --- | --- | --- | --- | --- | --- | --- | --- | --- | --- | --- | --- | --- | --- | --- | --- | --- | --- | --- | --- | --- | --- | --- | --- | --- | --- | --- | --- | --- | --- | --- | --- | --- | --- | --- | --- | --- | --- | --- | --- | --- | --- | --- | --- | --- | --- | --- | --- | --- | --- | --- | --- | --- | --- | --- | --- | --- | --- | --- | --- | --- | --- | --- | --- | --- | --- | --- | --- | --- | --- | --- | --- | --- | --- | --- | --- | --- | --- | --- | --- | --- | --- | --- | --- | --- | --- | --- | --- | --- | --- | --- | --- | --- | --- | --- | --- | --- | --- | --- | --- | --- | --- | --- | --- | --- | --- | --- | --- | --- | --- | --- | --- | --- | --- | --- | --- | --- | --- | --- | --- | --- | --- | --- | --- | --- | --- | --- | --- | --- | --- | --- | --- | --- | --- | --- | --- | --- | --- | --- | --- | --- | --- | --- | --- | --- | --- | --- | --- | --- | --- | --- | --- | --- | --- | --- | --- | --- | --- | --- | --- | --- | --- | --- | --- | --- | --- | --- | --- | --- | --- | --- | --- | --- | --- | --- | --- | --- | --- | --- | --- | --- | --- | --- | --- | --- | --- | --- | --- | --- | --- | --- | --- | --- | --- | --- | --- | --- | --- | --- | --- | --- | --- | --- | --- | --- | --- | --- | --- | --- | --- | --- | --- | --- | --- | --- | --- | --- | --- | --- | --- | --- | --- | --- | --- | --- | --- | --- | --- | --- | --- | --- | --- | --- | --- | --- | --- | --- | --- | --- | --- | --- | --- | --- | --- | --- | --- | --- | --- | --- | --- | --- | --- | --- | --- | --- | --- | --- | --- | --- | --- | --- | --- | --- | --- | --- | --- | --- | --- | --- | --- | --- | --- | --- | --- | --- | --- | --- | --- | --- | --- | --- | --- | --- | --- | --- | --- | --- | --- | --- | --- | --- | --- | --- | --- | --- | --- | --- | --- | --- | --- | --- | --- | --- | --- | --- | --- | --- | --- | --- | --- | --- | --- | --- | --- | --- |

**KMO: 0.67**

**% cumulative variance: 19.47%**

**Table D - Factor scores for men between 15 and 44 years of age**

| **ATC** | **Drugs** | **Factor1** | **Factor2** | **Factor3** |
| --- | --- | --- | --- | --- |
| **J** | **Antiinfectives for systemic use** | **0.78** | -0.12 | 0.09 |
| **N02B** | **Other analgesics and antipyretics** | **0.68** | 0.01 | 0.00 |
| **R05D** | **Cough suppressants, excluding combinations with expectorants** | **0.60** | -0.08 | 0.07 |
| **M01A** | **Antiinflammatory and antirheumatic products, non-steroids** | **0.57** | 0.07 | 0.00 |
| **R05C** | **Expectorants, excluding combinations with cough suppressants** | **0.51** | 0.04 | 0.26 |
| **A02B** | **Drugs for peptic ulcer and gastro-oesophageal reflux disease** | **0.38** | **0.37** | -0.11 |
| **N06A** | **Antidepressants** | -0.11 | **0.86** | 0.04 |
| **N05B** | **Anxiolytics** | -0.04 | **0.81** | 0.14 |
| **N05A** | **Antipsychotics** | -0.05 | **0.74** | -0.01 |
| **N03A** | **Antiepileptics** | 0.15 | **0.70** | -0.15 |
| **C10A** | **Lipid modifying agents, plain** | 0.00 | **0.36** | 0.04 |
| **R01A** | **Decongestants and other nasal preparations for topical use** | 0.07 | 0.03 | **0.74** |
| **R06A** | **Antihistamines for systemic use** | 0.02 | 0.04 | **0.70** |
| **R03A** | **Adrenergics, inhalants** | 0.05 | 0.00 | **0.61** |

**KMO: 0.67**

**% cumulative variance: 58.54%**

Table E - Factor scores for men between 45 and 64 years of age

| **ATC** | **Drug** | **Factor1** | **Factor2** | **Factor3** | **Factor4** |
| --- | --- | --- | --- | --- | --- |
| **B01A** | **Antithrombotic agents** | **0.91** | 0.03 | -0.03 | -0.07 |
| **C10A** | **Lipid modifying agents, plain** | **0.75** | 0.08 | -0.02 | -0.14 |
| **C01D** | **Vasodilators used in cardiac diseases** | **0.72** | 0.15 | -0.13 | 0.01 |
| **C07A** | **Beta blocking agents** | **0.71** | 0.07 | -0.06 | -0.09 |
| **C08C** | **Selective calcium channel blockers with mainly vascular effects** | **0.61** | -0.06 | -0.02 | 0.09 |
| **A10A** | **Insulins and analogues** | **0.56** | 0.00 | -0.18 | 0.13 |
| **A10B** | **Blood glucose-lowering drugs, excluding insulins** | **0.54** | -0.08 | -0.07 | 0.05 |
| **C03C** | **High-ceiling diuretics** | **0.46** | 0.22 | 0.24 | -0.31 |
| **C09A** | **ACE inhibitors, plain** | **0.43** | 0.04 | 0.03 | -0.04 |
| **A02B** | **Drugs for peptic ulcer and gastro-oesophageal reflux disease** | **0.39** | 0.29 | 0.13 | **0.32** |
| **C09C** | **Angiotensin II antagonists, plain** | **0.39** | -0.02 | 0.08 | -0.02 |
| **M04A** | **Antigout preparations** | **0.36** | -0.03 | 0.12 | 0.00 |
| **C09D** | **Angiotensin II antagonists, combinations** | **0.31** | -0.03 | -0.01 | 0.10 |
| **N06A** | **Antidepressants** | 0.01 | **0.77** | -0.03 | -0.11 |
| **N05A** | **Antipsychotics** | -0.20 | **0.75** | -0.03 | -0.08 |
| **N05B** | **Anxiolytics** | 0.13 | **0.73** | -0.03 | -0.08 |
| **N03A** | **Antiepileptics** | -0.03 | **0.61** | -0.06 | 0.15 |
| **N05C** | **Hypnotics and sedatives** | 0.11 | **0.61** | 0.04 | -0.07 |
| **A06A** | **Laxatives** | 0.07 | **0.45** | -0.13 | 0.27 |
| **A02A** | **Antacids** | 0.02 | **0.34** | 0.17 | 0.01 |
| **A03F** | **Propulsives** | -0.03 | **0.33** | 0.05 | 0.17 |
| **N02A** | **Opioids** | 0.02 | **0.33** | 0.02 | **0.33** |
| **R03A** | **Adrenergics, inhalants** | 0.06 | 0.00 | **0.85** | -0.24 |
| **R03B** | **Other drugs for obstructive airway diseases, inhalants** | 0.08 | 0.06 | **0.72** | -0.31 |
| **J** | **Antiinfectives for systemic use** | -0.01 | -0.05 | **0.63** | 0.26 |
| **R05C** | **Expectorants, excluding combinations with cough suppressants** | 0.01 | -0.04 | **0.62** | 0.18 |
| **R06A** | **Antihistamines for systemic use** | -0.05 | 0.00 | **0.58** | 0.04 |
| **R01A** | **Decongestants and other nasal preparations for topical use** | -0.03 | -0.06 | **0.56** | 0.09 |
| **H02A** | **Corticosteroids for systemic use, plain** | -0.01 | 0.14 | **0.53** | -0.04 |
| **N02B** | **Other analgesics and antipyretics** | 0.08 | 0.09 | **0.35** | **0.41** |
| **R05D** | **Cough suppressants, excluding combinations with expectorants** | 0.00 | -0.05 | **0.31** | **0.31** |
| **M01A** | **Antiinflammatory and antirheumatic products, non-steroids** | -0.05 | 0.11 | 0.24 | **0.54** |
| **M03B** | **Muscle relaxants, centrally acting agents** | -0.06 | 0.16 | 0.01 | **0.38** |
| **M02A** | **Topical products for joint and muscular pain** | 0.08 | 0.15 | 0.14 | **0.33** |
| **C03B** | **Low-ceiling diuretics, excluding thiazides** | 0.17 | -0.05 | -0.11 | **0.31** |
| D07A | Corticosteroids, plain | 0.13 | 0.00 | 0.17 | 0.20 |
| G04C | Drugs used in benign prostatic hypertrophy | 0.07 | 0.13 | 0.02 | 0.18 |
| S01X | Other ophthalmologicals | 0.12 | 0.12 | 0.08 | 0.15 |
| D01A | Antifungals for topical use | 0.02 | 0.21 | 0.19 | 0.13 |
| S01E | Antiglaucoma preparations and miotics | 0.17 | -0.01 | 0.02 | 0.11 |
| C09B | ACE inhibitors, combinations | 0.26 | -0.03 | 0.03 | -0.03 |

**KMO: 0.73**

**% cumulative variance: 39.23%**

**Table F - Factor scores for men over 64 years of age**

| **ATC** | **Drug** | **Factor1** | **Factor2** | **Factor3** |
| --- | --- | --- | --- | --- |
| **J** | **Antiinfectives for systemic use** | **0.65** | 0.02 | -0.11 |
| **R05C** | **Expectorants, excluding combinations with cough suppressants** | **0.60** | 0.02 | -0.08 |
| **H02A** | **Corticosteroids for systemic use, plain** | **0.55** | -0.10 | -0.08 |
| **R03A** | **Adrenergics, inhalants** | **0.54** | 0.00 | -0.05 |
| **R06A** | **Antihistamines for systemic use** | **0.49** | 0.00 | -0.08 |
| **N02B** | **Other analgesics and antipyretics** | **0.48** | 0.08 | 0.07 |
| **R03B** | **Other drugs for obstructive airway diseases, inhalants** | **0.44** | 0.04 | -0.04 |
| **R05D** | **Cough suppressants, excluding combinations with expectorants** | **0.42** | 0.03 | -0.11 |
| **S01C** | **Antiinflammatory agents and antiinfectives in combination** | **0.37** | 0.04 | -0.36 |
| **M01A** | **Antiinflammatory and antirheumatic products, non-steroids** | **0.37** | -0.06 | 0.02 |
| **A12A** | **Calcium** | **0.34** | -0.05 | 0.17 |
| **S01B** | **Antiinflammatory agents** | **0.33** | 0.15 | -0.48 |
| **S01A** | **Antiinfectives** | **0.32** | 0.11 | -0.24 |
| **N02A** | **Opioids** | **0.31** | 0.05 | 0.18 |
| **S01X** | **Other ophthalmologicals** | **0.31** | 0.12 | 0.00 |
| **A02B** | **Drugs for peptic ulcer and gastro-oesophageal reflux disease** | **0.39** | **0.41** | 0.20 |
| **B01A** | **Antithrombotic agents** | -0.08 | **0.89** | 0.13 |
| **C01D** | **Vasodilators used in cardiac diseases** | 0.03 | **0.67** | 0.00 |
| **C07A** | **Beta blocking agents** | -0.10 | **0.59** | -0.03 |
| **C10A** | **Lipid modifying agents, plain** | -0.06 | **0.58** | -0.10 |
| **C03C** | **High-ceiling diuretics** | 0.13 | **0.52** | 0.20 |
| **A10A** | **Insulins and analogues** | -0.05 | **0.47** | -0.03 |
| **C03D** | **Potassium-sparing agents** | 0.04 | **0.39** | 0.30 |
| **C09A** | **ACE inhibitors, plain** | -0.02 | **0.38** | 0.01 |
| **C08D** | **Selective calcium channel blockers with direct cardiac effects** | 0.09 | **0.33** | 0.11 |
| **C08C** | **Selective calcium channel blockers with mainly vascular effects** | -0.03 | **0.31** | -0.02 |
| **N04B** | **Dopaminergic agents** | -0.13 | -0.08 | **0.50** |
| **N05A** | **Antipsychotics** | 0.05 | 0.00 | **0.41** |
| **C01A** | **Cardiac glycosides** | -0.09 | 0.26 | **0.39** |
| **N06A** | **Antidepressants** | 0.14 | 0.10 | **0.39** |
| **A06A** | **Laxatives** | 0.19 | 0.09 | **0.37** |
| **C05C** | **Capillary stabilising agents** | 0.19 | -0.03 | **0.33** |
| **C05A** | **Agents for treatment of haemorrhoids and anal fissures for topical use** | 0.13 | -0.14 | **0.31** |
| N06B | Psychostimulants, agents used for ADHD and nootropics | 0.10 | 0.13 | 0.27 |
| N05C | Hypnotics and sedatives | 0.20 | 0.13 | 0.27 |
| L02A | Hormones and related agents | -0.01 | -0.06 | 0.26 |
| N03A | Antiepileptics | 0.23 | 0.11 | 0.25 |
| A03F | Propulsives | 0.22 | -0.04 | 0.25 |
| G04B | Other urologicals, including antispasmodics | 0.05 | 0.09 | 0.25 |
| N06D | Anti-dementia drugs | -0.14 | 0.11 | 0.23 |
| N05B | Anxiolytics | 0.23 | 0.19 | 0.23 |
| B03B | Vitamin B12 and folic acid | 0.18 | 0.01 | 0.22 |
| B03A | Iron preparations | 0.16 | 0.20 | 0.21 |
| M02A | Topical products for joint and muscular pain | 0.25 | 0.06 | 0.14 |
| C02C | Antiadrenergic agents, peripherally acting | -0.05 | 0.13 | 0.14 |
| M05B | Drugs affecting bone structure and mineralisation | 0.29 | -0.14 | 0.11 |
| G04C | Drugs used in benign prostatic hypertrophy | 0.18 | 0.02 | 0.09 |
| A02A | Antacids | 0.19 | -0.02 | 0.08 |
| N07C | Antivertigo preparations | 0.14 | -0.04 | 0.06 |
| C09C | Angiotensin II antagonists, plain | -0.01 | 0.22 | 0.03 |
| D01A | Antifungals for topical use | 0.22 | 0.07 | 0.03 |
| C04A | Peripheral vasodilators | 0.06 | 0.29 | 0.02 |
| D07A | Corticosteroids, plain | 0.24 | -0.04 | 0.02 |
| C09D | Angiotensin II antagonists, combinations | 0.02 | 0.06 | 0.01 |
| C03B | Low-ceiling diuretics, excluding thiazides | 0.07 | -0.03 | 0.01 |
| C01E | Other cardiac preparations | 0.07 | 0.23 | 0.01 |
| A10B | Blood glucose-lowering drugs, excluding insulins | -0.08 | 0.29 | -0.04 |
| C03E | Diuretics and potassium-sparing agents in combination | -0.05 | 0.15 | -0.04 |
| M04A | Antigout preparations | 0.06 | 0.29 | -0.10 |
| S01E | Antiglaucoma preparations and miotics | 0.18 | 0.09 | -0.12 |
| C09B | ACE inhibitors, combinations | 0.02 | -0.01 | -0.12 |
| R01A | Decongestants and other nasal preparations for topical use | 0.27 | 0.05 | -0.16 |
| S01F | Mydriatics and cycloplegics | 0.15 | 0.09 | -0.72 |

**KMO: 0.69**

**% cumulative variance: 21.25%**
